# Evaluation of the mindfulness-augmented “Trampoline” programme – a German prevention programme for children from substance-involved families tested in a cluster-randomised trial

**DOI:** 10.1186/s12889-019-6875-1

**Published:** 2019-05-14

**Authors:** Diana Moesgen, Katharina Ise, Janina Dyba, Michael Klein

**Affiliations:** German Institute of Addiction and Prevention Research, Catholic University of Applied Sciences, Woerthstrasse 10, 50668 Cologne, Germany

**Keywords:** Substance use, Children, Addiction, Family, Intervention, cRCT, At-risk children, Mindfulness, Mindfulness-based stress reduction, MBSR

## Abstract

**Background:**

Children of substance-abusing parents are at a substantial risk of developing substance-use and other mental disorders. Children involved in substance abuse – not diagnosed with substance-use problems but integrated in psychiatric treatment or youth welfare services – constitute a particular high-risk group that is in need of substance use prevention. Emerging evidence indicates that self-regulatory determinants of substance use and other mental disorders, particularly stress reactivity, are modifiable by mindfulness-based interventions, such as mindfulness-based stress reduction.

**Methods:**

In this ongoing cluster randomised-controlled trial, a mindfulness-augmented version of the modularised evidence-based “Trampoline” programme for children affected by parental substance use problems is evaluated in a sample of 420 children who are from substance-involved families, aged from 8 to 12 and receiving non-substance-specific care in psychiatric or youth welfare services. Larger effects on adaptive stress-coping strategies (primary outcome), internalising and externalising problem behaviours and distress due to parental substance use are expected compared to the standard “Trampoline”-programme version. Mindfulness components will be added and regularly practiced for 30 min in each validated “Trampoline” module. Moreover, the feasibility of mindfulness-based interventions in psychiatric care and youth welfare services for children suffering from emotional and behavioural problems will be investigated in this study.

**Discussion:**

Despite recruitment challenges, this study provides a unique opportunity to develop and test a promising addiction-specific, mindfulness-based intervention for a target group at risk, i.e. children from substance-involved families.

**Trial registration:**

The trial was registered in the German Clinical Trials Register on July 16th 2018 (trial registration number (TRN): DRKS00013533). Any important protocol modifications are to be reported immediately. Protocol version v.2.1, 15th April 2019.

## Background

### Background to the study

The negative impact of parental substance use on children has been documented by multiple studies and reviews, especially on children of alcoholics [1, 2]. Besides prenatal substance exposure that can have negative physical and developmental consequences [1, 3, 4], psychological effects may additionally impair the affected children’s development. For example, children and adolescents affected by parental substance use show and conduct higher rates of emotional disorders, such as anxiety disorders and depression [5–7], social behaviour disorders [8, 9], or hyperactivity disorders [10, 11].

With regard to substance-use problems, records of children with substance-abusing parents (CSP) have often shown an earlier onset of substance use [12], drunk experiences [13], increased binge drinking rates [14] and an elevated risk of developing substance-use disorders (SUD) at a younger age than comparable peers [15]. Overall, approximately 33 to 40% of the CSP develop SUD [16, 17]. SUD can be transmitted intergenerationally via several pathways, where genetic disposition, environmental factors as well as cognitive and psychological processes are relevant [18]. With regard to cognitive processes, CSP often have the same positive expectations about the effects of substance use as their parent [19]. As an important psychological factor, CSP later come to favour substance use as a maladaptive coping mechanism when experiencing stress and strain, having learned this strategy from the substance-using parent as a model [20].

Dysfunctional family characteristics may also have an impact on the maladaptive development of CSP. In their home environments, CSP are frequently exposed to psychological stress due to physical and psychological violence, volatile parenting, recurrent family conflicts [1, 21, 22] as well as a range of socioeconomic and health disadvantages resulting from their families’ financial problems and their parents’ complex health condition (e.g. psychiatric comorbidity) [23]. Length, type and frequency of exposure to parental substance-use and related parental behaviour have been seen as fundamental etiopathogenic factors for maladaptive developmental pathways [16]. Hence, it is important to offer early interventions to promote a healthy child development. Early prevention programmes are not only helpful on an individual level, but also in reducing societal costs related to delinquency, mental and physical disorders and child maltreatment [24–26].

Despite the urgent need, prevention programmes targeting CSP are still lacking in Germany, especially with respect to an evidence-base on effectiveness and practicability [27]; “Trampoline” is, by so far, the only one of its kind [28, 29]. Central topics of intervention are parental addiction and related problems as well as coping strategies (see below). This programme uses versatile modules that are suitable for the developing stages of 8–12-year-old boys and girls.

The prior randomised-controlled “Trampoline” trial demonstrated efficacy by comparing the manualised, psychoeducational preventive group intervention “Trampoline” with the results of a non-educational “fun and play” group of the same duration in a nationwide German sample of outpatient alcohol and drug treatment facilities; “Trampoline” has been proven to be effective in improving stress coping skills, reducing psychological distress due to parental substance addiction, improving psychological well-being as an aspect of quality of life and improving children’s self-concept and feelings of autonomy as well as the child-parent relationship [29]. Compared to the control group, “Trampoline” achieved a substantially more pronounced long-term reduction in psychological distress tested in a 6-month follow-up. Additionally, it demonstrated clear superiority over the control group regarding addiction-related knowledge. Manual adherence was good (84%), and children, parents and trainers showed high acceptance towards the programme [28, 30].

In sum, it can be concluded that “Trampoline” is effective for CSP. Nevertheless, this programme can still be improved with regard to significance of effect and relevant outcomes such as emotion regulation skills, an important element of stress management skills. Mindfulness training is a promising method to enhance children’s socioemotional resilience and the efficacy of existing prevention programmes in various psychological disorders and SUDs through imposing a positive impact on self-regulatory processes [31–33], including emotion regulation [34]. Mindfulness, defined as “systematic development of attention to present-moment experience with an attitude of accepting and non-judging” [35, 36], is expected to foster the ability to become more aware of habit-linked, affective states and bodily sensations and observe these experiences from a more detached and less reactive perspective rather than attempting to escape or distance oneself from unpleasant feelings and sensations [37, 38]. A recent review [34] demonstrates that mindfulness-based stress reduction (MBSR) is associated with improvements in emotion regulation, including recognition and management of emotions, emotional well-being, interpersonal relationships and stress reduction.

### Aims and objectives of the study

The aim of our ongoing study is to assess the effectiveness of a mindfulness-augmented version of the original “Trampoline”-programme (“Trampoline-Mind”) for children aged 8–12 years, have at least one substance-abusing parent and are suffering from emotional and/or behavioural problems. The effectiveness of the intervention will be tested in a multicentre cluster-randomised controlled trial with three points of measurement (pre, post and follow-up) while comparing it to the original “Trampoline”-programme and treatment-as-usual (TAU). It is hypothesised that “Trampoline-Mind” is feasible and effective for CSP and has had a positive impact on relevant predictors of SUD and other mental disorders. In detail, a mindfulness-augmented version of “Trampoline” is expected to improve children’s application of stress coping strategies in favour of problem-focused stress coping and constructive-palliative emotion regulation over avoidant stress coping and destructive-anger-related emotion regulation compared to the established “Trampoline”-programme. This suggests that CSP can benefit from integrating mindfulness components in the existing programme. As a secondary hypotheses, it is expected that “Trampoline-Mind” reduces distress due to parental substance use as well as internalising and externalising problem behaviours more effectively than the original “Trampoline” programme.

Moreover, by including mindfulness elements and using reliable and objective measures in a rigorous research design, this study allows the evidence on mindfulness-based interventions to be enlarged. Mindfulness training has an established evidence base, but only for adults [34]. Nevertheless, recent research has also supported the feasibility and efficacy of mindfulness-based techniques for children and adolescents [32, 39]. However, the current evidence is largely restricted to non-clinical settings, such as school settings [34], and it remains untested whether mindfulness-based interventions are effective in the prevention of SUD and related disorders among a highly vulnerable group such as CSP. Therefore, the study presented here will make use of a cluster-randomised-controlled trial longitudinal study design to establish profound evidence on the efficacy of the mindfulness-based “Trampoline-Mind” programme in a clinical and youth welfare setting, targeting the highly vulnerable group of CSP.

## Methods

### Study centres

German inpatient and outpatient psychiatric centres as well as inpatient youth welfare services were chosen as project facilities since they have access to the children affected by parental substance-use problems and showing first signs of psychological problems. The original “Trampoline”-programme has been delivered within facilities offering outpatient drug and alcohol counselling. Thus, “Trampoline-Mind” will not only augment the original version but also be tested within a new setting that is highly relevant to the target group.

### Trial design

The 9-session group programme “Trampoline-Mind” for children from substance-affected families is currently tested with regard to its effectiveness in a multicentre cluster-randomised trial (cRCT). The study presented here will compare the new “Trampoline-Mind” version (intervention group) to a) the original “Trampoline”-programme (control group I) with the same duration as “Trampoline-Mind” and b) a TAU-group (control group II). Both the intervention group and the control group I are delivered in the project facilities mentioned above. The programme to be delivered will be determined by randomisation which will be performed computer-based by the research team at Catholic University of Applied Sciences North Rhine-Westphalia. Control group II is a natural group that is not a part of the randomisation. Data from all three groups are gathered at three points: prior to intervention (T0), immediately after the intervention (T1) and six months after (T2). Last data collections for T2 are scheduled for January 2021.

The programme is designed for 8 to 12-year olds; exceptions can occasionally be made for 7- and 13-year-old children, depending on their developmental status. At baseline (T0), 420 children from 84 clusters are intended to enter the programme. Each cluster consists of an average size of five children.

**Fig. 1:**
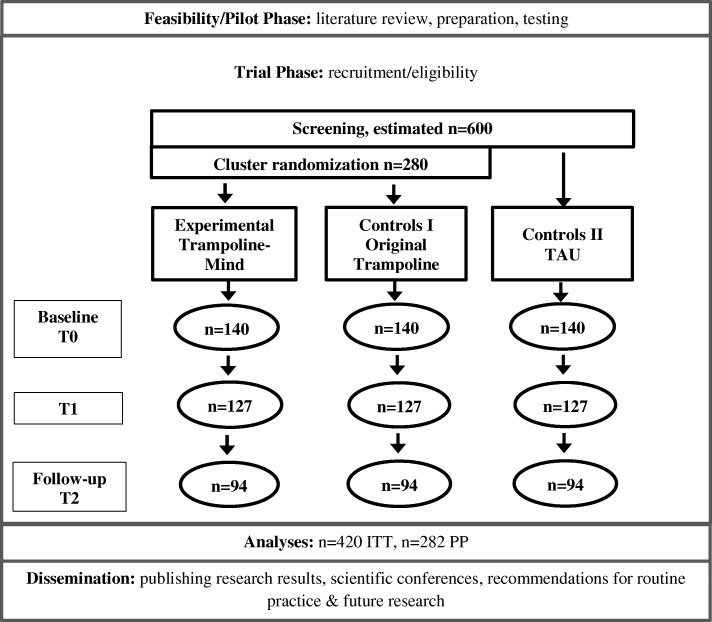
Procedure of the study

The procedure of this study is presented in Fig. 1.

### Interventions

The original “Trampoline”-programme, which control condition I receives, is a standardised, manualised group intervention that consists of nine modules for children and one module for parents (one part before the first child module and the other after the last child module). The envisaged group size is approximately five children, with every group moderated by two trained trainers. One module lasts 120 min, including a break matching the duration of the mindfulness-augmented modules. The original “Trampoline” modules will employ the following standard elements:

- Module 1 – getting to know each other
- Module 2 – self-worth: how I feel about myself
- Module 3 – alcohol and/or drug problems in my family
- Module 4 – knowledge: what I need to know about drugs and addiction
- Module 5 – handling difficult emotions
- Module 6 – self-efficacy: what I can do to solve problems
- Module 7 – learning new patterns of behaviour in my family
- Module 8 – what I can do to find help and support
- Module 9 – a positive good-bye

The original “Trampoline”-programme thus contains elements of psychoeducation, emotion regulation skills, strengthening of self-efficacy and self-worth, stress management and problem-solving strategies. All sessions follow the same structure and include recurring small rituals, as predictability is of major importance to children from substance-involved families.

The mindfulness-augmented version, “Trampoline-Mind”, which is delivered to the intervention group, maintains the structure, format and content of “Trampoline”, but adds 30 min of mindfulness-related exercises to each module. Core mindfulness dimensions will be included, such as present-moment awareness, body awareness and mindful movement, relaxation, mindfulness in dealing with emotions and thoughts, attention and non-judgmental acceptance, self-acceptance and self-compassion and kindness towards self and others. The development of the mindfulness exercises is based on a rigorous review of available evidence of mindfulness-based interventions for young people and according to the developmental needs and characteristics of children aged 8–12 years (e.g. cognitive abilities such as information processing, reasoning, language development, memory, attention span, knowledge of concepts and environmental requirements).

The parent module is the same for both the groups under study. It contains information and sensitisation on growing up in a substance-affected family, exercises on positive parenting, feedback on the course of the children’s groups and motivation for use of further help and support.

Both “Trampoline-Mind” and the original “Trampoline”-programme are delivered by trained trainers with a professional background in social work, psychology or similar.

No “Trampoline”-intervention is delivered to participants in control intervention II; this group will receive TAU only.

### Sample

Participants are CSP recruited by the participating study centres with the help of information materials provided by the research team at Catholic University of Applied Sciences North Rhine-Westphalia. Study centres inform participants and parents about the content and goal of the programme and study and screen participating CSP for eligibility. Inclusion criteria are:

1. children aged between 8 and 12 years (exceptions made for 7- and 13-year olds; see above) at T0,
2. children currently situated at an inpatient or outpatient psychiatric treatment or psychiatric day care or inpatient youth welfare service,
3. children exposed to parental substance use within last year (“parent” may also be a caregiver),
4. children either living with the substance-affected parent or having regular contact with him or her (at least once a month),
5. positive screening of parent on Alcohol Use Disorder Identification Test (AUDIT) [40] and/or Drug Use Disorder Identification Test (DUDIT) [41, 42] (and/or optional: positive screening of child on Children of Alcoholics Screening Test (CAST)) [43, 44],
6. sufficient mastery of the German language by both children and parents to participate in assessments and
7. informed written consent from both parents and children.

Children are excluded from the study if

1. they are diagnosed with or suspected of foetal alcohol spectrum disorder and/or cognitive impairment and/or severe conduct disorder or if
2. they have received any kind of addiction-specific treatment relevant for the study goals six months prior to the study or if
3. they have insufficient German language skills.

### Data collection

As mentioned above, data from all three groups are gathered at three points: at baseline, i.e. prior to intervention (T0), immediately after the intervention (T1) and six months after (T2).

With regard to measurements, age-adequate standardised instruments are used to assess outcome variables. Data gathered from CSP include e.g. socio-demographic data, stress level and coping strategies, emotion regulation, current parental substance use, relationship quality with the parent, addiction-related knowledge, health-related quality of life and mindfulness-related variables. In addition, parents (ideally both) or primary caregivers are asked to report on psychological stress, substance-use problems, relationship quality with the child, assertiveness regarding their own parenting competence, parenting style, child behaviour and mindfulness-related variables. An overview of measurements is presented in Table 1.

**Table 1:** Measurements

Target variable	Measure
Child	
Socio-demographic characteristics	Own development
Body Mass Index	Own development
Emotional and behavioural difficulties	SDQ-D (Strengths and Difficulties Questionnaire) (for children aged 11 or older only)
Excessive behaviours	Own development
Health-related quality of life	KIDSCREEN-27
Quality of relationship between parent and child	Own development: questions on a thermometer-scale in regard to closeness vs. distance and harmony vs. conflict
Parental drug and addiction problems	CAST-6 (Children of Alcoholics Screening Test)
Knowledge on alcohol, drugs, and substance use problems	Own development
Perceived stress	PSS-4 (Perceived Stress Scale)
Emotion Regulation	DERS (Difficulties in Emotion Regulation Scale)
Stress and coping	SSKJ 3–8 (Questionnaire for perception of stress and stress management in childhood and adolescence)
Mental distress caused by parental substance use	Own development
Seeking help	Own development
Experience with mindfulness-based interventions	Own development
Mindful attention awareness	MAAS-C (Mindful Attention Awareness Scale for children)
Treatment as usual	Own development
Parent	
Socio-demographic characteristics	Own development
Child emotional and behavioural difficulties	SDQ-D (Strengths and Difficulties Questionnaire; parent version)
Treatment as usual (child)	Own development
Self-confidence in parenting skills	FKE (Questionnaire for parental feelings of competence)
Parental Monitoring	Own development
Family functioning	Family APGAR
Quality of relationship between parents and child	Own development: questions on a thermometer-scale in regard to closeness vs. distance and harmony vs. conflict
Parental use of substance-related help	Own development
Mental health problems	SCL-K-9 (Symptom Checklist, short version)
Parental alcohol problems	AUDIT (Alcohol Use Disorders IdentificationTest)
Parental drug problems	DUDIT (Drug Use Disorders Identification Test)
Experience with mindfulness-based interventions	Own development
Mindful attention awareness	MAAS (Mindful Attention Awareness Scale)

In each condition, all participating children will receive a 10,00€ gift card as compensation for their participation at all points of measurement.

Besides analyzing data on the effectiveness of the “Trampoline-Mind” and “Trampoline”-programme, process evaluation data is collected as well. For this, trainers from the participating centres are asked to complete short questionnaires on adherence, group interaction and challenges encountered after each child and parent session. Moreover, trainers are asked to complete questionnaires on relevant characteristics such as structural data on their institution or professional background. In addition, process evaluation data from children and parents are gathered after each session. These data will be useful for analyzing e.g. the quality of manual adherence. Moreover, the data can help identifying potential moderating factors such as trainer qualification or group dynamics.

### Statistical analyses

The calculation of the sample size for this study (*n* = 420 children from 84 clusters) is based on a power analysis for detecting small effect sizes, using a two-sided test at alpha = 0.05 and a power of (1-beta) = 0.80, with a maximum dropout rate of 30%.

All data is entered manually. Analyses will be performed using the statistical software EpiData. Descriptive and inferential statistical analyses are conducted according to research questions.

### Ethical considerations

All participating study centres and parents received detailed written information on research goals, study procedures, data analyses and data reporting prior to participation. Written consent is obtained from all parents and children before entering the study and saved by the research team at the Catholic University of Applied Sciences North Rhine-Westphalia. Participants assigned to control condition I are asked to participate in the evidence-based original “Trampoline”-programme. Participants from control condition II have volunteered to participate in this group or are assigned to it for practical reasons with their informed written consent. They also receive TAU. All participating children and parents are encouraged to call the research group in case of arising questions or problems. In case of unexpected severe adverse events (SAE) the participating study centres are obliged to document and report these to the research team. It will then be decided on how to proceed (e.g. remove participants from intervention, initiate medical help).

All data processing will be conducted according to the current Data Protection Act. The research team at the Catholic University of Applied Sciences North Rhine-Westphalia has access to the final data set.

The ethics committee of the Catholic University of Applied Sciences North Rhine-Westphalia in Cologne, Germany, has approved all procedures.

A neutral data management security board (DSMB) and an external monitoring agency will supervise the adherence of the study to Good Clinical Practice (GCP) standards.

## Discussion

### Summary

Despite the existing evidence-based “Trampoline”-programme, there is a substantial need for the improvement of care targeting CSP. Even though “Trampoline” has been proven to be effective, the significance of effect were small, and emotion regulation skills can be enhanced further. These skills are key when it comes to coping with stress.

One promising idea for improving the programme is the integration of elements of mindfulness-based trainings, as these may be able to enhance CSP’s socioemotional resilience and self-regulatory processes [31–33], including emotion regulation [34].

Hence, a mindfulness-augmented version of the original “Trampoline”-programme (“Trampoline-Mind”) for CSP aged 8–12 years was developed and tested in a multicentre cRCT with three points of measurement (pre, post and follow-up) while comparing it to the original “Trampoline”-programme and TAU. It is expected that participants in the “Trampoline-Mind” will benefit from the integration mindfulness components into the “Trampoline” programme by demonstrating enhanced stress regulation skills compared to the participants from the original “Trampoline” and the TAU condition. This study will also enlarge the evidence on mindfulness-based interventions for children and adolescents in clinical and youth welfare settings.

### Implications

The intervention and its study design have several strengths: first, “Trampoline-Mind” is based on an evidence-based intervention tailored to the needs of CSP. It both considers theoretical foundations and practical experiences from the work with CSP. Second, the intervention can be delivered easily by professionals due to the self-explanatory trainer’s manual. “Trampoline-Mind” thus may be regarded as a pragmatic programme that can be used in clinical and youth welfare settings as well. Third, because of the strict separation between trainers and programme evaluators, bias is reduced. A further reduction of bias is achieved by gathering not only data form children’s self-reports but also from their parents’ point of view. Fourth, by conducting a 6-month follow-up, sleeper effects can be detected, and the stability of effects uncovered in the post-measurement can be tested. Fifth, the effects of “Trampoline-Mind” are compared to both the original “Trampoline”-programme and TAU. Therefore, specific effects of mindfulness-based elements and addiction-specific contents can be identified.

Study results will be disseminated within the scientific community via journal publications and presentations.

### Limitations

Due to the volatile nature of substance-involved families and high fluctuations the clinical or youth welfare setting, it might become challenging to motivate CSP to run through the programme entirely and/or reach them to participate in follow-up measurements. Also, many parents with SUD will not let their children participate in the programme because of denial or feelings of shame and guilt, thereby creating a selection effect in the sample. Moreover, CSP may be reluctant to participate because of the fear of stigmatisation by other children residing in their study centre. All reasons for rejections and dropouts are to be documented and will be analysed carefully.

Because of these recruitment issues, this study – which had been originally planned as a randomised-controlled study – has been transformed into a cRCT. It became clear early on that cooperating study centres will not be able to perform two parallel groups, delivering both the “Trampoline-Mind” and the original “Trampoline”-programme simultaneously, as they did not expect to reach a sufficient number of children at the same time. Thus, the two programmes will be delivered successively. The programme to be delivered will be determined at random.

In case of further recruitment challenges, outpatient alcohol and drug treatment facilities can be integrated as additional study centres. These facilities have close contact to parents with SUD and, sometimes, offer programmes for CSP, too. By adding this outpatient setting, a comparison of different accesses to CSP can be made.

At last, it is desired to compare the effects of the programme with a naturalistic sample of children from substance-affected families not receiving any intervention. However, this attempt would exceed the study’s resources.

## Abbreviations

AUDIT: Alcohol Use Disorder Identification Test
CAST: Children of alcoholics screening test
cRCT: Cluster-randomised trial
CSP: Children of substance-abusing parents
DSMB: Data management security board
DUDIT: Drug Use Disorder Identification Test
GCP: Good Clinical Practice
MBSR: Mindfulness-based stress reduction
SAE: Severe adverse event
SUD: Substance use disorder
TAU: Treatment as usual
